# Anterior insular cortex activity to emotional salience of voices in a passive oddball paradigm

**DOI:** 10.3389/fnhum.2014.00743

**Published:** 2014-09-22

**Authors:** Chenyi Chen, Yu-Hsuan Lee, Yawei Cheng

**Affiliations:** ^1^Institute of Neuroscience, National Yang-Ming UniversityTaipei, Taiwan; ^2^Department of Rehabilitation, National Yang-Ming UniversityYilan, Taiwan; ^3^Department of Education and Research, Taipei City HospitalTaipei, Taiwan

**Keywords:** mismatch negativity (MMN), magnetoencephalography (MEG), anterior insular cortex (AIC), emotional salience

## Abstract

The human voice, which has a pivotal role in communication, is processed in specialized brain regions. Although a general consensus holds that the anterior insular cortex (AIC) plays a critical role in negative emotional experience, previous studies have not observed AIC activation in response to hearing disgust in voices. We used magnetoencephalography to measure the magnetic counterparts of mismatch negativity (MMNm) and P3a (P3am) in healthy adults while the emotionally meaningless syllables *dada*, spoken as neutral, happy, or disgusted prosodies, along with acoustically matched simple and complex tones, were presented in a passive oddball paradigm. The results revealed that disgusted relative to happy syllables elicited stronger MMNm-related cortical activities in the right AIC and precentral gyrus along with the left posterior insular cortex, supramarginal cortex, transverse temporal cortex, and upper bank of superior temporal cortex. The AIC activity specific to disgusted syllables (corrected *p* < 0.05) was associated with the hit rate of the emotional categorization task. These findings may clarify the neural correlates of emotional MMNm and lend support to the role of AIC in the processing of emotional salience already at the preattentive level.

## Introduction

Mismatch negativity (MMN) has recently been used as an index of the salience of emotional voice processing (Schirmer et al., 2005; Cheng et al., 2012; Fan et al., 2013; Hung et al., 2013; Fan and Cheng, 2014; Hung and Cheng, 2014). MMN reflects the early saliency detection of auditory stimuli regarding stimulus discrimination based on the perceptual processes of physical features (Pulvermüller and Shtyrov, 2006; Thönnessen et al., 2010). Considering that the anterior insular cortex (AIC) plays a critical role in negative emotional experience (Craig, 2002, 2009), particularly in perceiving disgust, and magnetoencephalography (MEG) could complement the spatiotemporal dynamics in a passive auditory oddball paradigm, we proposed the AIC activation with respect to emotional MMN.

The AIC is a polysensory cortex involved in the awareness of bodily sensations and subjective feelings (Craig, 2002, 2009). Perceiving a disgusting odor, disgusted faces, and imagining feeling disgust have consistently activated the AIC (e.g., Phillips et al., 1997, 2004; Adolphs et al., 2003; Krolak-Salmon et al., 2003; Wicker et al., 2003; Jabbi et al., 2008). Menon and Uddin (2010) suggested that the AIC is a key region of the emotional salience network that integrates external stimuli with internal states to guide behaviors. However, previous studies have failed to identify the AIC activation associated with disgusted vocal expressions (Phillips et al., 1998).

MEG enables non-invasive measurements of neural activity with sufficient spatial resolution and excellent temporal resolution. MMN/its magnetic equivalent (MMNm), and P3a/P3am, can be elicited using a passive auditory oddball paradigm in which participants engage in a task and must ignore the stimuli that are presented in a random series, with one stimulus (standard) occurring more frequently than the other stimuli (deviant). P3a/P3am is associated with involuntary attention switches for sound changes (Alho et al., 1998). As a preattentive change detection index, MMN/MMNm can reflect N-methyl-D-aspartate receptor function (Näätänen et al., 2011), which mediates sensory memory formation and emotional reactivity in various neuropsychiatric disorders (Campeau et al., 1992; Barkus et al., 2010). MMNm elicited by emotional (happy and angry) deviants in an oddball paradigm can reflect early stimulus processing of emotional prosodies (Thönnessen et al., 2010). Recent studies have indicated that, in addition to being used as an index of the acoustic features of sounds, such as frequency, duration, and phonetic contents (e.g., Ylinen et al., 2006; Horvath et al., 2008), MMN can also be used as an index of the salience of emotional voices (Cheng et al., 2012; Fan et al., 2013; Hung et al., 2013; Fan and Cheng, 2014; Hung and Cheng, 2014).

Various manners have been used for the acoustic control of emotional voices. For example, scrambling voices enables the amplitude envelope to be preserved (Belin et al., 2000). In one study, simple tones synthesized from the strongest formant of the vowel were used as the control stimuli in the mismatch paradigm (Čeponienė et al., 2003). In another studies, physically identical stimuli were presented as both standards and deviants (Schirmer et al., 2005, 2008). Because no single acoustic parameter can fully explain strong neural responses to emotional prosodies (Wiethoff et al., 2008), the present study, using the same theorems as Belin et al. (2000) did, involved employing two stringent sets of acoustic control stimuli, simple tones and complex tones, to control the temporal envelope and core spectral elements of emotional voices [spectral centroid (fn) and fundamental frequency (f0)], respectively.

To elucidate the neural correlates underpinning the emotional salience of voices, we measured MMNm and P3am in a passive auditory oddball paradigm while presenting the neutrally, happily, and disgustedly spoken syllables *dada* to young adults. We hypothesized that, if the AIC is involved in the preattentive processing of emotional salience, then AIC activation would be observed in the source distribution of MMNm, in accordance with the auditory cortices and early response latencies. If AIC activation is specific to voices, MMNm in response to acoustic attributes, i.e., simple and complex tone deviants, would not elicit the AIC activation. If neurophysiologic changes can guide behaviors (Menon and Uddin, 2010), then people exhibiting stronger AIC activation in response to hearing emotional syllables are expected to perform more favorably in emotional recognition. Furthermore, because men and women might engage in dissimilar neural processing of emotional stimuli (Hamann and Canli, 2004), the gender factor was introduced into the analyses.

## Materials and methods

### Participants

Twenty healthy participants (10 men), aged 18–30 years (mean ± *SD*: 22 ± 1.9), underwent MEG recording and structural MRI scanning after providing written informed consent. The study was approved by the ethics committee in National Yang-Ming University and conducted in accordance with the Declaration of Helsinki. One person was excluded from data analysis because of motion artifacts. All participants were right-handed without hearing or visual impairments. They had no neurological and psychiatric disorders. Participants received monetary compensation for their participation.

### Auditory stimuli

The stimulus material consisted of three categories: emotional syllables, simple tones, and complex tones. For the emotional syllables, a young female speaker produced the syllables *dada* with two sets of emotional (happy and disgusted) prosodies and one set of neutral prosodies. Within each type of emotional or neutral prosodies, the speaker produced the *dada* syllables more than ten times to enable validation. Sound Forge 9.0 and Cool Edit Pro 2.0 were used to edit the syllables so that they were equally long (550 ms) and loud (max: 62 dB, mean: 59 dB).

Each syllable set was rated for emotionality on a 5-point Likert-scale by a total of 120 listeners (60 men). For the disgusted set, listeners classified each stimulus from *extremely disgusted* to *not disgusted at all*. For the happy set, listeners classified from *extremely happy* to *not happy at all* and for the neutral set, listeners classified from *extremely emotional* to *not emotional at all*. Emotional syllables that were consistently identified as the extremely disgusted and happy (i.e., the highest ratings) as well as the most emotionless (i.e., the lowest rating) were used as the stimuli. The Likert-scale (mean ± *SD*) of happy, disgusted, and neutral syllables were 4.34 ± 0.65, 4.04 ± 0.91, and 2.47 ± 0.87, respectively.

Although firmly controlling the spectral power distribution may result in the loss of temporal flow associated with formant contents in voices (Belin et al., 2000), the synthesizing the temporal envelope and the core spectral elements of voices should enable the maximal control of the spectral and temporal features of vocal and corresponding non-vocal sounds (Schirmer et al., 2007; Remedios et al., 2009). In order to create a set of stimuli that retain acoustical correspondence with the emotional syllables, we synthesized simple and complex tones by using Praat (Boersma, 2001) and MATLAB (The MathWorks, Inc., Natick, MA, USA). Using a sine waveform, we extracted the fundamental frequencies (f0) and the spectral centroid (fn) of each original syllable to produce complex and simple tones, respectively (Supplementary Figures S1, S2). The lower end of the spectrogram at each time point determined the fundamental frequency (f0). For the complex tones, the f0 over time was extracted to preserve the pitch contour. For the simple tones, the spectral centroid (fn), indicating the center of mass of the spectrum, was extracted to reflect the brightness of sounds. The original syllable envelope then multiplied the extracted frequencies. Hence, to control temporal features, three categories (emotional syllables, complex tones, and simple tones) were assigned to have identical temporal envelopes. To control spectral features, complex tones retained the f0 whereas simple tones retained the fn of emotional syllables. The length (550 ms) and loudness (max: 62 dB; mean: 59 dB) of all stimuli were controlled.

### Procedures

During MEG recording, participants lay in a magnetically shielded chamber and watched a silent movie with subtitles while the task-irrelevant vocally spoken or synthesized stimuli were presented. To ensure that the auditory stimuli were sufficiently irrelevant, participants attentively watched the movie and answered questions regarding the movie content after data recording.

Three sessions (emotional syllables, complex tones, and simple tones) were conducted. The session order was pseudorandomized among participants. In the emotional session, neutral syllables set as the standard (S), and happy and disgusted syllables designed as two isometric deviants (D1, D2) followed the oddball paradigm. During the complex and simple sessions, we applied an identical oddball paradigm for the corresponding synthesized tones so that relative acoustic features among S, D1, and D2 were controlled across all three categories. Each session consisted of 800 standards, 100 D1s, and 100 D2s. A minimum of two standards was presented between any two deviants. The successive deviants were always diverse. The stimulus onset asynchrony was 1200 ms.

After MEG recording, participants performed a forced-choice emotional categorization task. While listening to the forty-five stimuli, including five Ss, five D1s, and five D2s of each stimulus category, participants identified each emotional characteristics as one of three types (emotionless, happy, or disgusted) in a self-paced manner. The chance level was 33.33% based on three alternatives.

### Apparatus and recordings

The data were recorded by using a 157-channel axial gradiometer whole-head MEG system (Kanazawa Institute of Technology, Kanazawa, Japan). Prior to data acquisition, the locations of five head position indicator coils attached to the scalp and several additional scalp surface points were recorded with respect to fiduciary landmarks (nasion and two preauricular points) by using a 3-D digitizer, which digitized each participant's head shape and localized the position of the participant's head inside the MEG helmet. Data were collected at a sampling frequency of 1 kHz. Participants kept their heads steady during MEG recording. The head-shape and head-position indicator locations were digitized at the onset of recording and were later used to coregister the MEG coordinate system with the structural MRI of each participant. Structural MR images were acquired on a 3 T Siemens Magnetom Trio-Tim scanner using a 3D MPRAGE sequence (TR/TE = 2530/3.5 ms, FOV = 256 mm, flip angle = 7°, matrix = 256 × 256, 176 slices/slab, slice thickness = 1 mm, no gap).

### MEG preprocessing and analysis

In offline processing the MEG data, we applied a low-pass filter at 20 Hz (Luck, 2005) and reduced the noise using the algorithm of time-shifted principle component analysis (de Cheveigné and Simon, 2007; Hsu et al., 2011). The MEG data were then epoched for each trial type by time locking the stimulus onsets at 100-ms prestimulus intervals and 700-ms poststimulus intervals. Epochs with a signal range exceeding 1.5 fT at any channel were excluded from the averaging and subsequent statistical analyses, in which the deviant-stimulus averages were calculated based on at least 90 trials per participants. The amplitudes of averaged MEG response waveforms were measured with respect to a 100-ms prestimulus baseline.

### Event-related fields (ERF)

For amplitude and latency analyses, we used the Isofield Contour Map to identify the channels with the strongest signal in the direction. Because head position variation might unequally contribute to the differential activity observed at individual sensors, we created a composite map by grand-averaging nine conditions, where three D1s, three D2s and three Ss of three categories were pooled together. For the ERF difference, the difference maps [disgusted MMNm (D2-S); happy MMNm (D1-S)] were averaged for each category. Based on the composite maps of each ERF component, we selected the four clusters with the strongest signal in the direction. The amplitudes of the sensory ERF peaks (N1m and P2m), MMNm and P3am were measured as an average within a 60-ms window centered at each participant's individual peak latencies. P3am was defined as the component immediately following MMNm, peaking at 300-500 ms. Two-tailed *t* tests were used to determine the statistical presence (difference from 0 fT) of ERF peaks related to the stimuli.

Statistical analysis involved Three-Way mixed ANOVAs with two within-subject factors: category (emotional syllables, complex tones, or simple tones) and stimulus [neutral (S), happy (D1), or disgusted (D2)] and one between-subject factor: gender (males vs. females). The dependent variables were the amplitudes and latencies of each component. Bonferroni test was conducted only when preceded by significant effects.

### MEG source analysis

The structural MR images were processed using FreeSurfer (CorTechs Labs, La Jolla, CA and MGH/HMS/MIT Athinoula A. Martinos Center for Biomedical Imaging, Charleston, MA) to create a cortical reconstruction of each brain. Minimum-norm estimates (MNEs) (Hämäläinen and Ilmoniemi, 1994) were computed from combined anatomical MRI and MEG data by using the MNE toolbox (MGH/HMS/MIT Athinoula A. Martinos Center for Biomedical Imaging, Charleston, MA). For inverse computations, the cortical surface was decimated to 5000–10,000 vertices per hemisphere. We used the boundary-element model method to compute the forward solution, which was an estimate of the magnetic field at each MEG sensor resulting from the activity at each of the vertices. The forward solution was then employed to create the inverse solution, which enabled identifying the spatiotemporal distribution of any activity over sources, that most accurately account for each participant's average MEG data. The noise covariance matrix was estimated according to the prestimulus baselines of the individual trials. Only the components of activation that were in a direction normal to the cortical surface were retained in the minimum-norm solution. The MNE results were then converted into dynamic statistical parameter maps (dSPM), which measured the noise-normalized activation at each source and enabled several standard minimum-norm calculations inaccuracies to be avoided (Dale et al., 2000).

To test whether the evoked response significantly differed between conditions, the problem of multiple comparisons was addressed by conducting a cluster-level permutation test across space. For each cortical location within each region of interest (ROI), a paired-samples *t* value was computed for testing the deviant-standard contrast or the contrast between two deviants (*p* = 0.05). We then selected all of the samples for which this *t* value exceeded an a priori threshold (uncorrected *p* < 0.05). Finally, the selected samples were clustered according to spatial adjacency. By clustering neighboring cortical locations that exhibited the same effect, we addressed the multiple comparisons problem while considering the dependency of the data. Cortical dipoles were considered to be neighbors if the distance between them was less than 12 mm. A sample was included in the cluster only when there were at least two neighboring samples in space.

### Regions of interests (ROI) and source-specific time-course extraction

**Figure 1:**
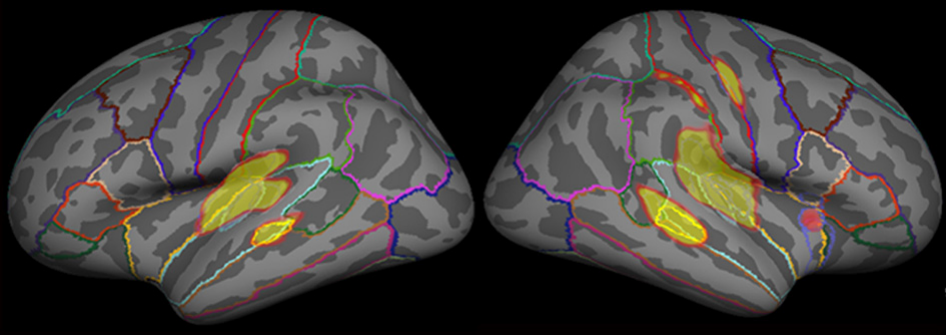
**The grand averaged functional map evoked by emotional syllables**. The functional map of dSPM solutions estimated for each category (emotional syllables, complex, and simple tones) was superimposed onto a reconstructed anatomical criterion where the vertices were optimally parceled out using the gyral-sulcal patterns. We overlaid the grand averaged functional map in response to emotional syllables onto the common reconstructed cortical sphere as an example.

The cortical surface of each participant was normalized onto a standard brain supported by FreeSurfer, and the dSPM solutions of all participants were subsequently averaged so that they could be used in the defined regions and time windows of interest. Considering the fact that the trial number contributed considerably to the inverse source estimation, we selected only the standard exactly preceding the deviant to estimate cortical activity The dSPM solutions estimated for the standards, which immediately preceded D1 and D2, were then averaged to represent the cortical activity for standard sound processing. To further qualitatively clarify the underlying neural correlates of MMNm and P3am, a functional map, using inclusive masking to display significant dSPM activation for either deviant (D1 or D2), was used to select the ROIs for all of the stimulus categories. Specifically, the grand averaged functional maps evoked by D1 and D2 were overlaid onto a common reconstructed cortical sphere, respectively. The ROIs were drawn along the border of functional maps as well as the anatomical criterion where the vertices were optimally parceled using the gyral-sulcal patterns (Fischl et al., 1999; Sereno et al., 1999; Leonard et al., 2010). The ROIs for D1 and D1 were then combined to form an inclusive mask displaying significant dSPM activations for either deviant (D1 or D2). Then, the dSPM time courses were extracted from the predetermined ROIs after their amplitudes were measured and calculated as described previously (Figure 1).

## Results

### Behavioral performance

**Figure 2:**
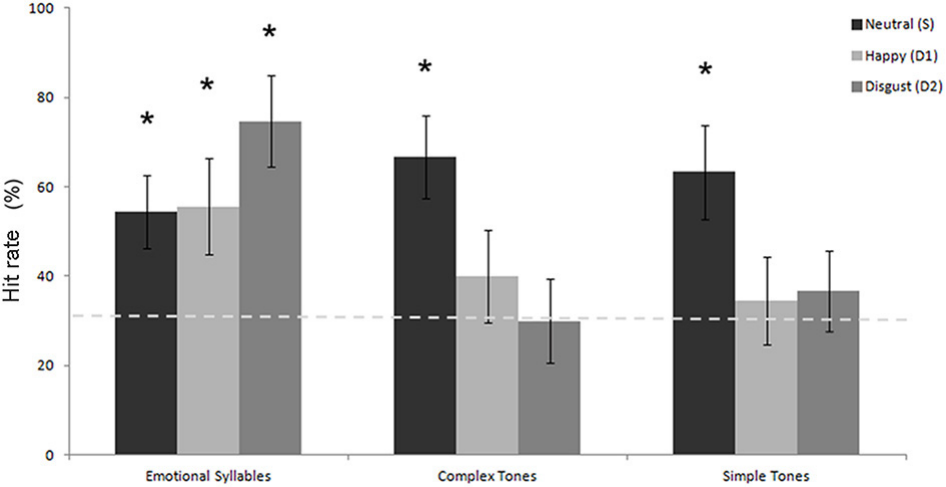
**Hit rate in the emotional categorization task**. The emotional syllables exhibited more favorable performance than did the complex and simple tones. Only the emotional syllables attained above-chance hit rates for each emotion. The asterisk (^*^p < 0.05) indicates that the hit rate is statistically higher than the chance level (dashed line).

Table 1 shows the performance on the emotional categorization task. A Three-Way mixed ANOVA targeting categories (emotional syllables, complex tones, or simple tones) and stimulus (neutral, happy, or disgusted) as the within-subject variables and gender (male vs. female) as the between-subject variable was computed in terms of the hit rate. The category effect [*F*_(2, 32)_ = 4.90, *p* = 0.01] and the interaction between category and stimulus [*F*_(4, 64)_ = 2.80, *p* = 0.03] were significant (Figure 2). No significance was observed regarding gender and gender-related interaction. The emotional syllables exhibited more favorable performance than did the complex (*p* = 0.031) and simple (*p* = 0.005) tones. *Post-hoc* comparisons showed that the neural- relative to emotional-derived tones exerted more favorable performance in the complex [Neutral- > Happy-derived tones: *t*_(17)_ = 1.9, Cohen's *d* = 0.70, one-tailed *p* = 0.035; Neutral- > Disgusted-derived tones: *t*_(17)_ = 2.5, Cohen's *d* = 0.92, one-tailed *p* = 0.01] and simple tones [*t*_(17)_ = 1.8, Cohen's *d* = 0.67, one-tailed *p* = 0.04; *t*_(17)_ = 1.8, Cohen's *d* = 0.67, one-tailed *p* = 0.04], but this pattern was not observed in the emotional syllables (*p* = 0.11; *p* = 0.94). Only the emotional syllables exerted above-chance hit rates (>33.33%) in all emotions, rather than the complex and simple tones, indicating emotional neutrality of acoustic controls.

**Table 1:** **Behavioral performance**.

**Emotional categorization task**	**Mean ± *SD***		
	**Emotional syllables**	**Complex tones**	**Simple tones**
Accuracies (%)	61.5 ± 28.8	48.5 ± 20.4	44.8 ± 15.1
Response times (ms)	798 ± 422	791 ± 297	731 ± 252

### Sensory ERF

Each stimulus type of each category reliably elicited an N1-P2 complex, which is typically obtained in adults during fast stimulus presentation (Supplementary Table S1 and Figure S3) (Näätänen and Picton, 1987; Pantev et al., 1988; Tremblay et al., 2001; Shahin et al., 2003; Ross and Tremblay, 2009).

Statistical analyses for each identified cluster on the Isofield Contour Map revealed that N1m had the category effect for the cluster over the right anterior region [*F*_(2, 36)_ = 5.33, *p* = 0.009] and P2m had the category effect for the clusters over the left and right posterior regions [*F*_(2, 36)_ = 15.03, *p* < 0.001; *F*_(2, 36)_ = 13.65, *p* < 0.001]. *Post-hoc* analyses indicated that the emotional syllables elicited stronger N1m than did the complex tones (*p* = 0.005) and simple tones (*p* = 0.02) for the cluster over the right anterior region. For those over the left and right posterior regions, the simple tones elicited stronger P2m amplitudes than did the emotional syllables (left: *p* = 0.03; right: *p* = 0.02) and complex tones (*p* = 0.01; *p* < 0.001). There was no significance for gender and gender-related interaction.

### MMNm

All deviant stimuli of each category elicited MMNm significantly. Statistical analyses for each identified cluster on the Isofield Contour Map revealed that there were significant interactions between category and stimulus over the left [*F*_(2, 36)_ = 4.73, *p* = 0.015] and right posterior [*F*_(2, 36)_ = 5.05, *p* = 0.012] clusters. In the left posterior cluster, *post-hoc* analyses showed that the stimulus effect where disgusted (D2) relative to happy (D1) MMNm was larger in amplitudes was present in the emotional syllables (*p* = 0.004), but none was detected in the simple tones (*p* = 0.27) and complex tones (*p* = 0.41). In the right posterior cluster, the stimulus effect was found in the emotional syllables (*p* < 0.001) and simple tones (*p* = 0.005). Gender and gender-related interaction were not significant (*p* >0.05).

### MMNm-related cortical activities

**Figure 3:**
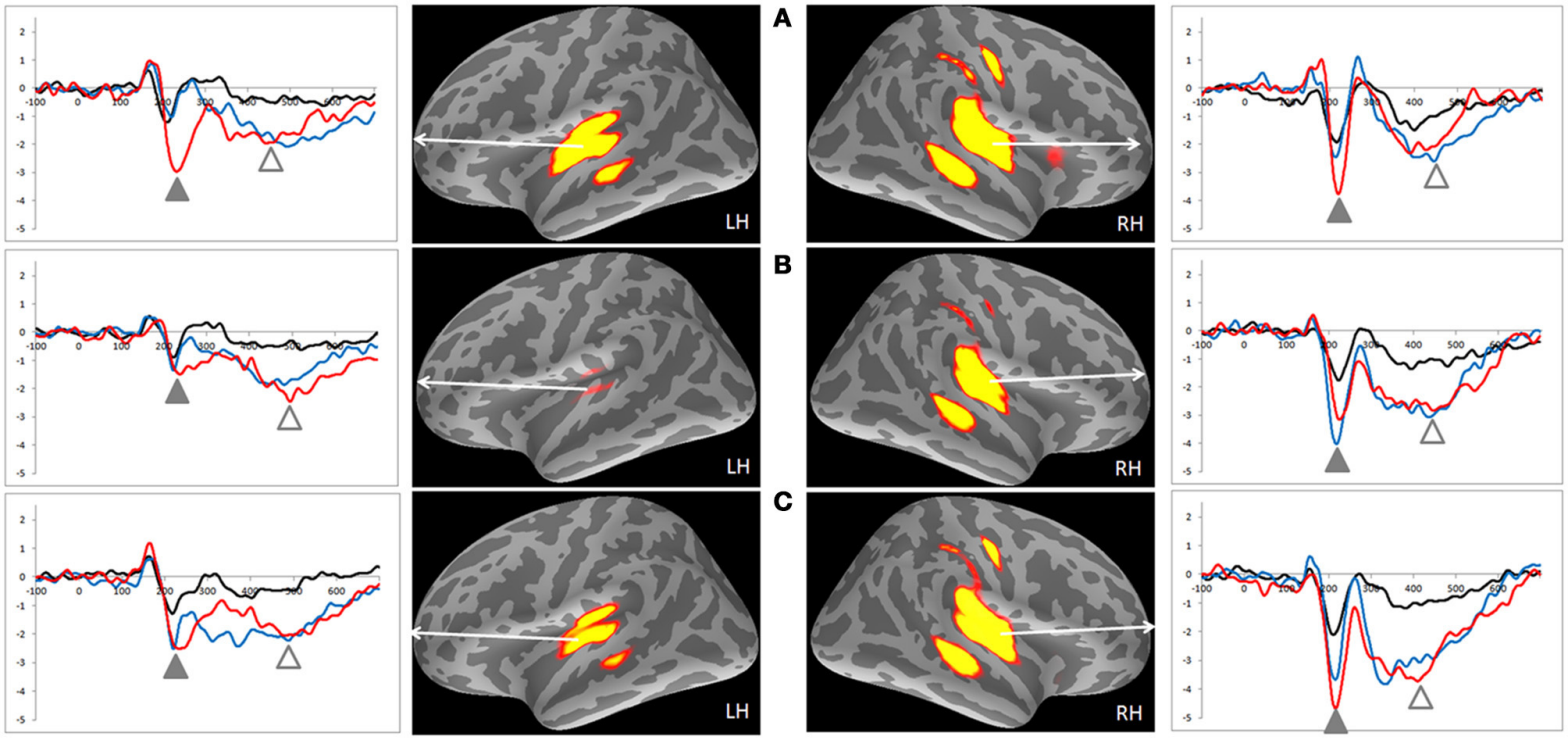
**MMNm-related cortical activities and source-specific (dSPM) time courses of ROIs in the emotional syllables and complex and simple tones**. **(A)** Emotional syllables. **(B)** Complex tones. **(C)** Simple tones. Positive dSPM values indicate the current flowing outward from the cortical surface, whereas negative values indicate the current flowing inward. Grand average (*n* = 19 participants) time courses of the mean estimated current strength for happy (D1, blue line), disgusted (D2, red line) ad neutral syllables (S, black line) were extracted from primary auditory cortex. The functional map shows significant dSPM activation in the early and late time window, indicated by solid and empty triangles, respectively.

**Figure 4:**
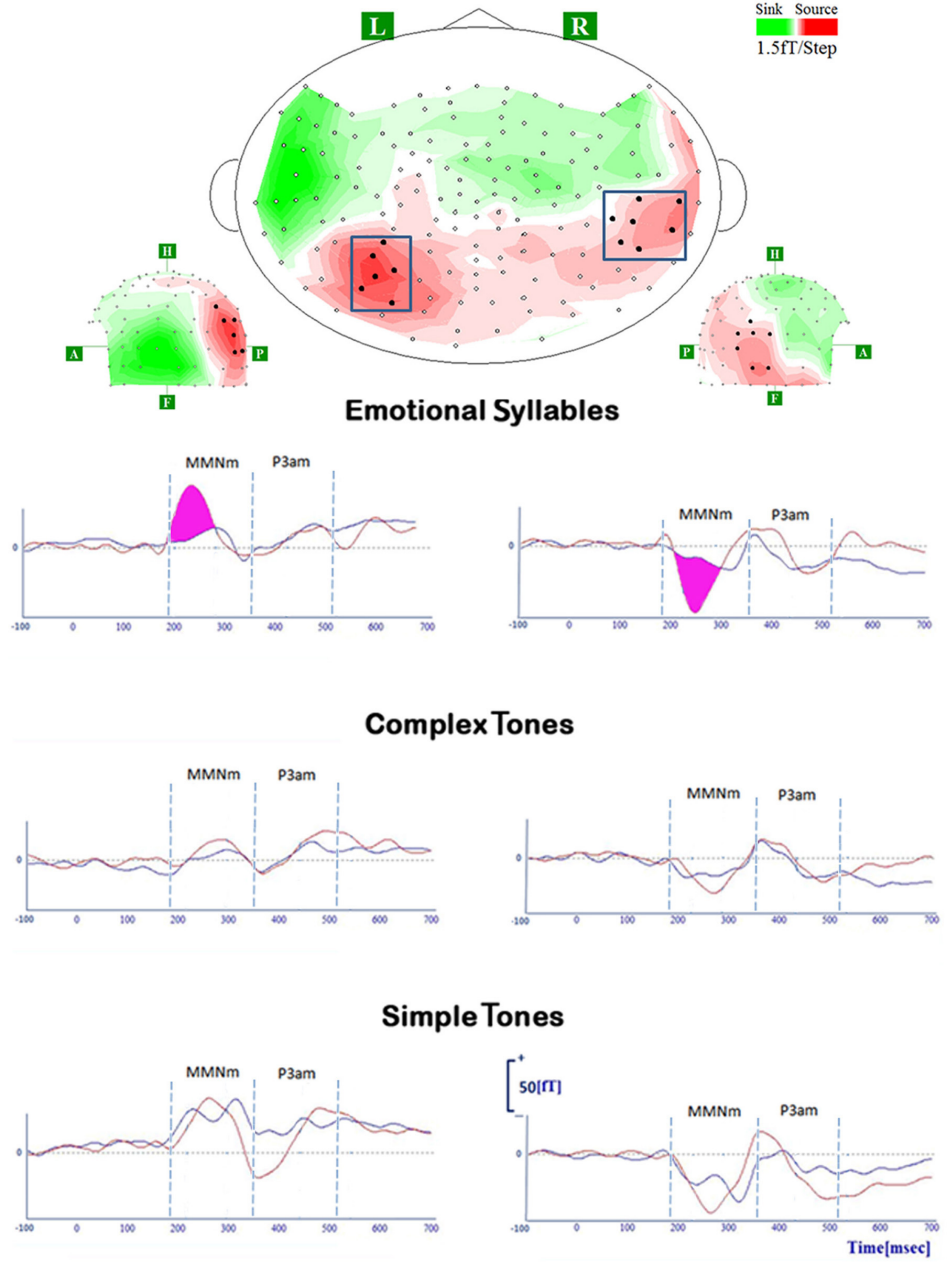
**MMNm and P3am in response to deviant stimuli of emotional syllables, complex tones, and simple tones**. All deviant stimuli of each category (emotional syllables, complex, and simple tones) consistently elicit MMNm and P3am [red line: disgusted MMNm (D2-S); blue line: happy MMNm (D1-S)]. For the sake of argument, the grand-average whole-head topography was derived from emotional syllables at the peak latency of MMNm (265 ms). The magenta area indexes the emotional salience processing, as shown by significant differences between disgusted MMNm and happy MMNm.

Table 2 lists the peak latencies used for analyzing source-specific amplitudes for each ROI. Statistical analyses, using a Three-Way mixed ANOVA targeting category (emotional syllables, complex tones, or simple tones) and stimulus [neutral (S), happy (D1), or disgusted (D2)] as the within-subject variables and gender (males vs. females) as the between-subject variable for each ROI, revealed that the brain regions exhibiting an interaction between the category and stimulus were the right AIC [*F*_(4, 68)_ = 3.35, *p* = 0.015], right precentral gyrus [*F*_(4, 68)_ = 5.54, *p* = 0.001], left supramarginal cortex [*F*_(4, 68)_ = 3.03, *p* = 0.023], upper and lower bank of superior temporal sulcus (uSTS and lSTS) [*F*_(4, 68)_ = 2.78, *p* = 0.03; *F*_(4, 68)_ = 3.41, *p* = 0.03] together with the left posterior insular cortex (PIC) [*F*_(4, 68)_ = 5.09, *p* = 0.006], left and right transverse temporal cortex [*F*_(4, 68)_ = 4.71, *p* = 0.002; *F*_(4, 68)_ = 3.02, *p* = 0.02] (Figure 3). Gender and gender-related interaction were non-significant. *Post-hoc* tests indicated that the processing of emotional salience (Figure 4), as indicated by stronger cortical activities for disgusted syllables relative to happy syllables (D2 > D1: FDR corrected *p* < 0.05) occurred in the right AIC [*F*_(2, 34)_ = 7.84, *p* = 0.002] and precentral gyrus [*F*_(2, 34)_ = 7.98, *p* = 0.005] along with the left PIC [*F*_(2, 34)_ = 5.55, *p* = 0.008], supramarginal cortex [*F*_(2, 34)_ = 6.71, *p* = 0.004], transverse temporal cortex [*F*_(2, 34)_ = 8.95, *p* = 0.001], and uSTS [*F*_(2, 34)_ = 8.49, *p* = 0.001].

**Table 2:** **MMNm-related cortical activities**.

**Regions of interest (ROI)**	**L/R**	**MNI coordinates**			**Peak latencies (ms; mean ± *SD*)**			***Post-hoc FDR < 0.05***		
		***X***	***Y***	***Z***	**Emotional**	**Complex**	**Simple**	**Emotional**	**Complex**	**Simple**
Anterior insular cortex (AIC)	R	30	16	4	240 ± 24			D2>S,D2>D1		
Posterior insular cortex (PIC)	L	−31	−22	10	241 ± 22			D2>D1		
Superior temporal sulcus, upper bank (uSTS)	L	−50	−26	2	244 ± 27	248 ± 25	248 ± 27	D2>S,D2>D1	D2>S	D2>S
Supramarginal cortex	L	−42	−24	22	240 ± 23	245 ± 20		D2>D1	D2>S	
Transverse temporal cortex	L	−45	−22	7	245 ± 25	248 ± 24		D2>D1	D2>S	
Transverse temporal cortex	R	49	−19	4		241 ± 23	233 ± 20		D1>S	D2>S
Superior temporal sulcus, upper bank (uSTS)	L	−50	−27	0	244 ± 26		248 ± 26	D2>S,D2>D1		D2>S
Precentral gyrus	R	44	−16	36	230 ± 23		232 ± 18	D2>D1		D1>S,D2>S
Posterior insular cortex (PIC)	R	34	−16	0			233 ± 22			D2>S
Superior temporal sulcis, lower bank (lSTS)	R	48	−28	2		240 ± 23			D1>S	

*S, standard (neutral); D1, happy; D2, disgusted*.

**Figure 5:**
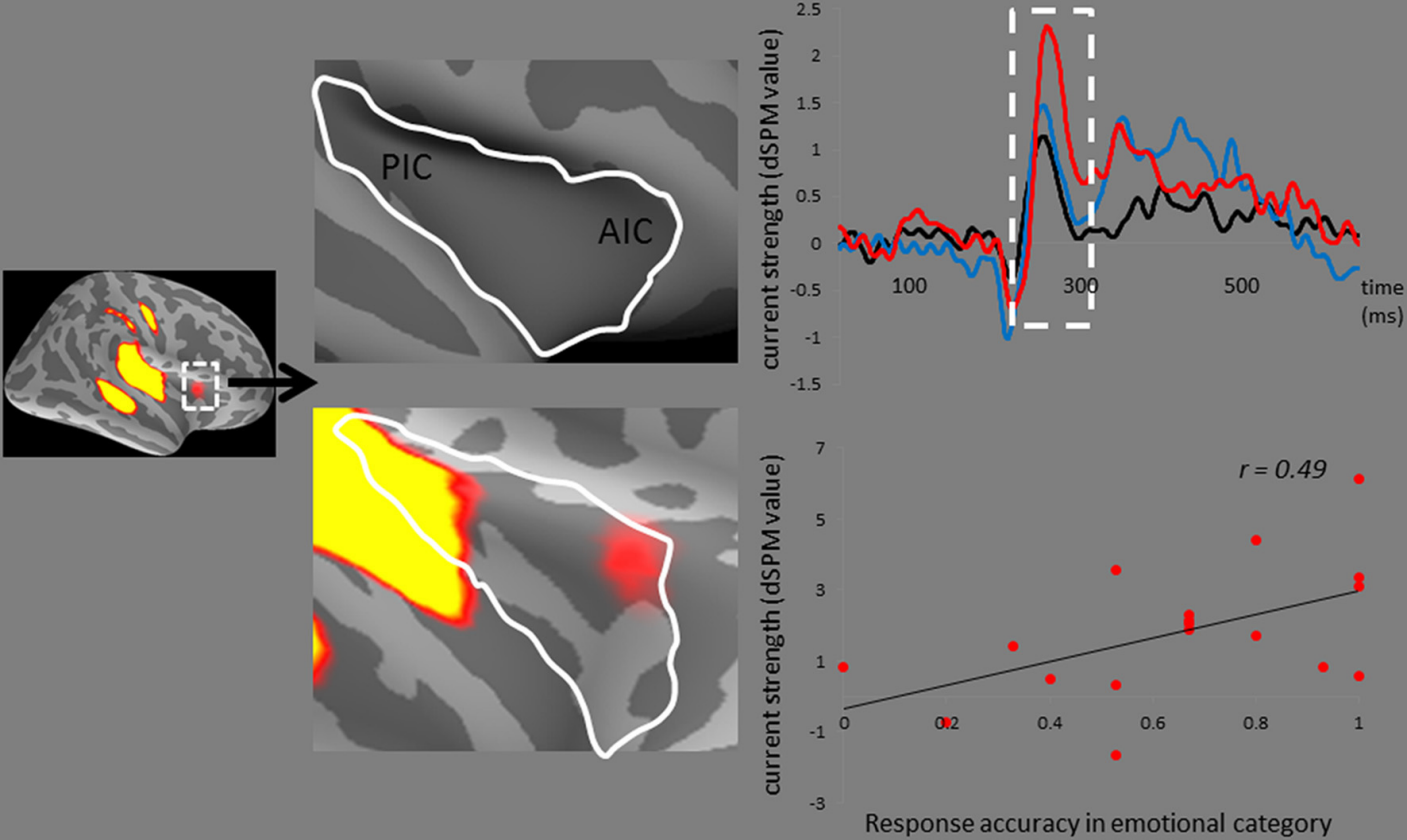
**The anterior insular cortex activity in response to disgusted syllables**. Disgusted deviants exclusively elicited the activation in the AIC, as indicated by MMNm-related cortical activities. Grand average (*n* = 19 participants) time courses of the mean estimated current strength were extracted from the right AIC (red line: disgust, D2; blue line: happy, D1; black line: standard, S). The AIC activation for disgusted deviants and the hit rates in the emotional categorization task were positively correlated [*r*_(18)_ = 0.49, *p* = 0.036].

In addition, the right AIC activities, which surpassed the dSPM criterion, specifically responded to the disgusted syllables (Figure 5). The correlation analysis revealed that the MMNm-related AIC activities were associated with the hit rates for emotional syllables in the emotional categorization task [*r*_(18)_ = 0.49, *p* = 0.036]. Participants exhibiting larger amplitudes in the right AIC activation triggered by disgusted syllables were likely to perform better in the emotional categorization task.

### P3am

Paired *t*-tests used to determine the statistical presence (difference from 0 fT/cm) indicated that all deviants from each category elicited P3am, temporally following MMNm (Supplementary Table S2). The ANOVA model on P3am amplitudes for each identified cluster on the Isofield Contour Map, did not find any significance in the category, stimulus, gender, and their related interaction (all *p* > 0.05).

### P3am-related cortical activities

P3am and MMNm had similar brain sources and manifested as two contiguous peaks in the dSPM source-specific time course (Figure 3 and Table 3). Statistical analyses on P3am-related cortical activities used a Three-Way mixed ANOVA targeting category (emotional syllables, complex tones, or simple tones) and stimulus [neutral (S), happy (D1), or disgusted (D2)] as the within-subject variables and gender (male vs. female) as the between-subject variable. The brain regions exhibiting the stimulus effect included the right supramarginal cortex [*F*_(2, 34)_ = 25.37, *p* = 0.032], uSTS [*F*_(2, 34)_ = 23.48, *p* = 0.018], lSTS [*F*_(2, 34)_ = 30.94, *p* = 0.006], and posterior superior temporal sulcus (pSTS) [*F*_(2, 34)_ = 17.44, *p* = 0.004], as well as the left transverse temporal cortex [*F*_(2, 34)_ = 16.67, *p* = 0.002], supramarginal cortex [*F*_(2, 34)_ = 12.00, *p* = 0.008], PIC [*F*_(2, 34)_ = 16.25, *p* = 0.006], uSTS [*F*_(2, 34)_ = 14.81, *p* = 0.003], and lSTS [*F*_(2, 34)_ = 26.42, *p* = 0.001]. None of ROIs reached any interaction between the category and stimulus. Gender and gender-related interaction were non-significant.

**Table 3:** **P3am-related cortical activities**.

**Regions of interest**	**L/R**	**MNI coordinates**			**Peak latencies (ms; mean ± SD)**			***Post-hoc FDR < 0.05***		
		***X***	***Y***	***Z***	**Emotional**	**Complex**	**Simple**	**Emotional**	**Complex**	**Simple**
Supramarginal cortex	R	46	−24	20	383 ± 36	389 ± 46	379 ± 35	D1>S	D1>S,D2>S	D1>S,D2>S
Superior temporal sulcus, upper bank (uSTS)	R	52	−29	9	363 ± 34	380 ± 43	370 ± 38	D1>S,D2>S	D1>S,D2>S	D1>S,D2>S
Superior temporal sulcus, lower bank (lSTS)	R	48	−28	2	386 ± 32	392 ± 40	380 ± 37	D1>S,D2>S	D1>S,D2>S	D1>S,D2>S
Supramarginal cortex	L	−42	−24	22	408 ± 44	419 ± 41	409 ± 52	D1>S,D2>S	D1>S,D2>S	D1>S,D2>S
Transverse temporal cortex	L	−45	−22	7	415 ± 43	420 ± 42	411 ± 49	D1>S,D2>S	D1>S,D2>S	D1>S,D2>S
Superior temporal sulcus, upper bank (uSTS)	L	−50	−26	2	405 ± 46	409 ± 45	411 ± 51	D1>S,D2>S	D1>S,D2>S	D1>S,D2>S
Posterior insular cortex (PIC)	L	−31	−22	10	412 ± 42	432 ± 42	412 ± 42	D1>S,D2>S	D1>S,D2>S	D1>S,D2>S
Superior temporal sulcus, lower bank (lSTS)	L	−50	−27	0	420 ± 45	427 ± 51	414 ± 48	D1>S,D2>S	D1>S,D2>S	D1>S,D2>S
Posterior superior temporal sulcus (pSTS)	R	43	−38	1	364 ± 35	366 ± 36	360 ± 37	D1>S,D2>S	D1>S,D2>S	D2>S

*S, neutral; D1, happy; D2, disgusted*.

## Discussion

Although the AIC plays a critical role in the negative experience of emotions (Craig, 2002, 2009; Menon and Uddin, 2010), including disgust (e.g., Phillips et al., 1997, 2004; Adolphs et al., 2003; Krolak-Salmon et al., 2003; Wicker et al., 2003; Jabbi et al., 2008), previous studies have not observed AIC activation in response to hearing disgusted voices. It thus leaves a room for more research to clarify whether AIC activation is specific to disgust or, alternatively, reflects general aversive arousal in response to negative emotions. In contrast to the predicted correlation between AIC activation and disgust recognition, we determined that the AIC activation predicted the performance of emotion recognition in general within the emotional category. This may be partially attributed to the functional role of the AIC in salience processing (Seeley et al., 2007; Sridharan et al., 2008; Menon and Uddin, 2010; Legrain et al., 2011). One MEG study on disgusted faces reported that early insular activation occurs at approximately 200 ms after the stimulus onset of emotionally arousing stimuli, regardless of valence, whereas the later insular response (350 ms) differentiates disgusted from happy facial expressions (Chen et al., 2009). Accordingly, AIC activation occurred in response to disgusted voices at approximately 250 ms in our study. The AIC is a brain region underpinning error awareness and saliency detection (Sterzer and Kleinschmidt, 2010; Harsay et al., 2012). This passive oddball study required no target detection. The AIC activation, which surpassed the dSPM criterion, specifically responded to disgusted syllables rather than happy syllables. Participants exhibiting stronger AIC activities were likely to have higher hit rats in the emotional categorization task (please see Figure 5). In addition, disgusted relative to happy syllables exhibited stronger MMN-related cortical activities, lending support for the notion that disgusted relative to happy voices might be more acoustically salient (Banse and Scherer, 1996; Simon-Thomas et al., 2009; Sauter et al., 2010).

Using MEG in a passive auditory oddball paradigm, we demonstrated the involvement of AIC in the preattentive perception of disgusted voices. MMNm-related AIC activation was specific to disgusted syllables, but not happy syllables. In addition, acoustically matched simple and complex tones did not activate the AIC in the same manner. Participants who exhibited stronger MMNm-related AIC activations were more prone to obtaining higher hit rates in the emotional categorization task. The involvement of AIC in emotional MMNm appears to be consistent between genders.

The MMNm response was sensitive to the positive and negative valence of emotional voices, as indicated by stronger amplitudes elicited by disgusted syllables than by happy syllables. Particularly, in the left hemisphere, the emotional salience processing was specific to voices rather than their acoustic attributes (see Figure 4). This should not be surprising because affective discrimination beyond acoustical distinction emerges early in the neonatal period (Cheng et al., 2012). Hearing angry and fearful syllables relative to happy syllables elicited stronger MMN (Schirmer et al., 2005; Fan et al., 2013; Hung et al., 2013; Fan and Cheng, 2014; Hung and Cheng, 2014). From an evolutionary perspective, disgust, an aversive emotion, exhibits a negativity bias that elicits stronger responses than neutral events do (Lange, 1922; Huang and Luo, 2006).

MMNm-related AIC activation may reflect emotional salience at the preattentive level. The significance of this finding lies in the lack of similar findings in the auditory modality by previous studies, despite the widespread evidence of the involvement of AIC in the experience of negative emotions. Particularly, attentively hearing disgusted voices did not activate the AIC (Phillips et al., 1998), indicating that AIC may be involved in the preattentive processing of disgusted voices. Theoretically, the passive auditory oddball paradigm should be the optimal approach for probing the preattentive processing of emotional voices because MMN can indicate the neural activity in a comatose or deep-sleeping brain (Kotchoubey et al., 2002). Although a silent movie presentation is unable to guarantee the lack of awareness to auditory stimuli, limited attention resources can indeed modulate the neurophysiological processing of emotional stimuli (Pessoa and Adolphs, 2010). We do not assert that the preattentive processing of emotional salience of voices was only dominated by the AIC. Through the AIC, the cortical-subcortical interactions for coordinating the function of cortical networks might be attributed to the neural mechanism underpinning the evaluation of the biological significance of affective voices.

The PIC exhibited stronger MMNm-related cortical activities for disgusted syllables relative to happy syllables, possibly involved in the representation of emotional salience. The PIC that has been functionally identified as the portion of the extended auditory cortex responded preferentially to vocal communication sounds (Remedios et al., 2009). The salient sensory information would reach the multimodal cortical areas, such as the PIC, directly from the thalamus, bypassing primary sensory cortices. This direct thalamocortical transmission is parallel to the modality-specific processing of stimulus attributes via the transmission from the thalamus to the relevant primary sensory cortices (Liang et al., 2013). In the present study, failing to observe any activation in the thalamus and anterior cingulate cortex in every condition could be attributed to the stringent statistic dSPM criterion as well as the absence of the target detection in a passive oddball task. The insular cortex, including AIC and PIC, can monitor the salience (appetitive and aversive) and integrate with the stimulus effect on the state of the body (Deen et al., 2011).

In addition, the left transverse temporal cortex, a part of primary auditory cortex, was sensitive to the processing of emotional salience [disgust (D2) > happy (D1)]. This finding supports that vocal emotional expression might be processed beyond the right hemisphere, being anchored within sensory, cognitive, and emotional processing systems at an early auditory discrimination stage (Schirmer and Kotz, 2006). The activation of the precentral gyrus observed across all three categories may reflect general attention and memory enhancement during information processing (Chen et al., 2010).

Importantly, the present study identified several future areas of inquiry. First, familiarity might potentially confound the effect of affective modulation observed here. Simple and complex tones are less familiar than emotional voices, and it might be impossible to categorize synthesized tones in relation to natural speech sounds. Second, using a pseudoword, such as *dada*, as an example might limit the generalizability for emotion representation. By using non-linguistic emotional vocalizations (Fecteau et al., 2007), additional studies are needed to verify whether the passive oddball paradigm is optimal for detecting emotional salience. Third, based on three alternatives, we defined the hit rate as the number of hits divided by the total number of trials at each stimulus category. This study did not control false alarm rates with the traditional approaches within the framework of Signal Detection Theory [SDT: d′ = Z (hit rate) − Z (false alarm rate)]. The performance of acoustic controls showed the skewed distribution, in which participants tended to classify the emotional-derived tones as neutral. Accordingly, a higher hit rate for neutral-derived tones than happy-/disgusted-derived tones might potentially violate the assumption that acoustic controls are emotionless. On the other hand, the sum of the hit rate for emotional syllables in the emotional categorization task, which prevents false alarms, exhibited a better prediction to the AIC activation than did the hit rate specific for disgust syllables. The hit rate of 75% for disgusted syllables and 55% for happy syllables corroborated existing findings in the identification of prototypical disgust and pleasure vocal burst (Simon-Thomas et al., 2009). However, only 55% being the hit rate for happy syllables has to be interpreted with caution. Future research should include the pre-evaluation of voices not only on the target emotion but also on other valence scales.

This MEG study clearly demonstrated the right AIC activation in response to disgusted deviance in a passive auditory oddball paradigm. The MMNm-related AIC activity was associated with the emotional categorization performance. The findings may clarify the neural correlates of emotional MMN and support that the AIC is involved in the processing of emotional salience at the preattentive level.

### Conflict of interest statement

The authors declare that the research was conducted in the absence of any commercial or financial relationships that could be construed as a potential conflict of interest.
