# Supplementary material for: Anterior insular cortex activity to emotional salience of voices in a passive oddball paradigm
Source: Front Hum Neurosci. 2014 Sep 22;8:743. doi: 10.3389/fnhum.2014.00743 (PMC4193252; doi:10.3389/fnhum.2014.00743)
Supplement: Supplementary file 1 [file DataSheet1.DOC]

**Supplementary Materials**

**Table s1: Sensory event-related fields (ERF) of the four identified clusters on the Isofield Contour Map.**

**The mean amplitudes (mean ± SE) and peak latencies (mean ± SD) of the N1m and P2m respond to the stimuli.**

|  |  | Amplitude (fT) | |  | Latency (ms) | |
| --- | --- | --- | --- | --- | --- | --- |
| Area | Category | N1m | P2m |  | N1m | P2m |
| Left  Anterior | Emotional syllables | 18.1 (4.4)^†^ | -31 (6.3)^‡^ |  | 167 (18) | 224 (33) |
|  | Complex tones | 11 (3.4)^†^ | -25 (6.1)^†^ |  | 159 (24) | 227 (28) |
|  | Simple tones | 17.7 (2.5)^‡^ | -36.7 (7.7)^‡^ |  | 154 (35) | 219 (31) |
| Right  Anterior | Emotional syllables | -18.9 (3)^‡^ | 40.3 (9.4)^‡^ |  | 162 (15) | 219 (17) |
|  | Complex tones | -6.5 (3)^*^ | 43.1 (8)^‡^ |  | 159 (18) | 224 (24) |
|  | Simple tones | -7.3 (3.3)^*^ | 50 (7.2)^‡^ |  | 158 (11) | 217 (11) |
| Left  Posterior | Emotional syllables | -29.5 (4.6)^‡^ | 36.9 (9.4)^†^ |  | 170 (25) | 228 (30) |
|  | Complex tones | -20.8 (3.5)^‡^ | 32.6 (8.9)^†^ |  | 178 (26) | 235 (32) |
|  | Simple tones | -24.5 (4.7)^‡^ | 57.8 (12.6)^‡^ |  | 162 (21) | 235 (23) |
| Right  Posterior | Emotional syllables | 21.5 (3.4)^‡^ | -41.4 (6.3)^*^ |  | 178 (13) | 220 (13) |
|  | Complex tones | 16.3 (3.3)^‡^ | -32.8 (6.1)^*^ |  | 181 (12) | 237 (30) |
|  | Simple tones | 16.5 (3.1)^‡^ | -64.5 (7.7)^‡^ |  | 169 (24) | 237 (27) |

Paired t-tests were used to determine the statistical presence (difference from 0 fT/cm) of the *ERFs*:

*****, *P* <0.05; †, *P* < 0.01; ‡, *P* < 0.001.

**Table s2: MMNm and P3am of the identified clusters on the posterior of the Isofield Contour Map.**

**The mean amplitudes (mean ± SE) and peak latencies (mean ± SD) of MMNm and P3am respond to the stimuli.**

|  |  | Amplitude (fT) | | Latency (ms) | |
| --- | --- | --- | --- | --- | --- |
| Area | Stimulus class | MMNm | P3am | MMNm | P3am |
| Left  Posterior | Emotional D1 (happy) | 8.8 (3.6)^*^ | 12.1 (5.5)^*^ | 278 (39) | 497 (31) |
|  | Emotional D2 (disgust) | 44.2 (10.3)^‡^ | 14.6 (7.8)^ms^ | 261 (37) | 489 (35) |
|  | Complex D1 (happy-derived) | 11.8 (5.5)^*^ | 15 (5.2)^†^ | 271 (31) | 465 (21) |
|  | Complex D2 (disgust-derived) | 20.7 (9.9)^*^ | 24.5 (10.7)^*^ | 273 (29) | 486 (33) |
|  | Simple D1 (happy-derived) | 35.1 (8.2)^‡^ | 27.1 (6.3)^‡^ | 274 (42) | 475 (40) |
|  | Simple D2 (disgust-derived) | 43.1 (7.7)^‡^ | 30 (6)^‡^ | 268 (27) | 493 (34) |
| Right  Posterior | Emotional D1 (happy) | -12.6 (4.2)^†^ | -19.6 (6.2)^†^ | 275 (45) | 478 (47) |
|  | Emotional D2 (disgust) | -59.3 (10.6)^‡^ | -20.4 (8.4)^*^ | 249 (22) | 479 (28) |
|  | Complex D1 (happy-derived) | -20.5 (5.4)^†^ | -15.6 (7.8)^ms^ | 264 (34) | 469 (33) |
|  | Complex D2 (disgust-derived) | -27.5 (8)^†^ | -13.7 (12.1) | 265 (18) | 476 (33) |
|  | Simple D1 (happy-derived) | -27 (6.3)^‡^ | -18.2 (7.2)^*^ | 276 (48) | 473 (37) |
|  | Simple D2 (disgust-derived) | -48.8 (7.9)^‡^ | -34.1 (9)^†^ | 263 (26) | 481 (44) |

Paired t-tests were used to determine the statistical presence (difference from 0 fT/cm) of the MEG peaks:

*****, *P* <0.05; †, *P* < 0.01; ‡, *P* < 0.001;ms, marginal-significant *P* < 0.1.

**Figure s1: Oscillogram (upper panel) and spectrogram (lower panel) of original sounds and the complex tones.**

**a. Original sounds**

**b. The complex tones**

The pitch contour (blue line) presents the flow of the concentration of energy at the lower end of the spectrogram (F0) at each time point. The complex tones retained the f0 flow as well as the same temporal envelope of the original sounds.


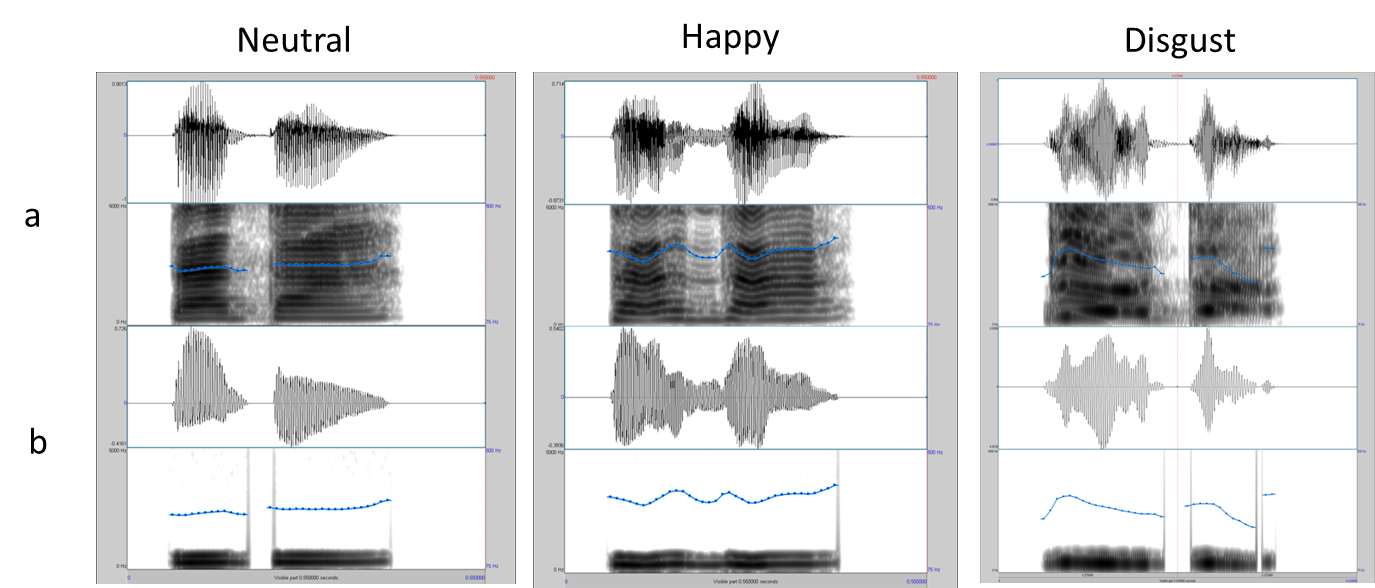


**Figure s2: Oscillogram and spectrogram of original sounds and the simple tones.**

**a. Oscillogram of original sounds**

**b. Spectrogram of original sounds**

**c. Sound envelop of both original sounds and the simple tones**

**d. Oscillogram of the simple tones**

**e. Spectrogram of the simple tones**

The simple tones retained the spectral centroid (fn) as well as the same temporal envelope of the original sounds.


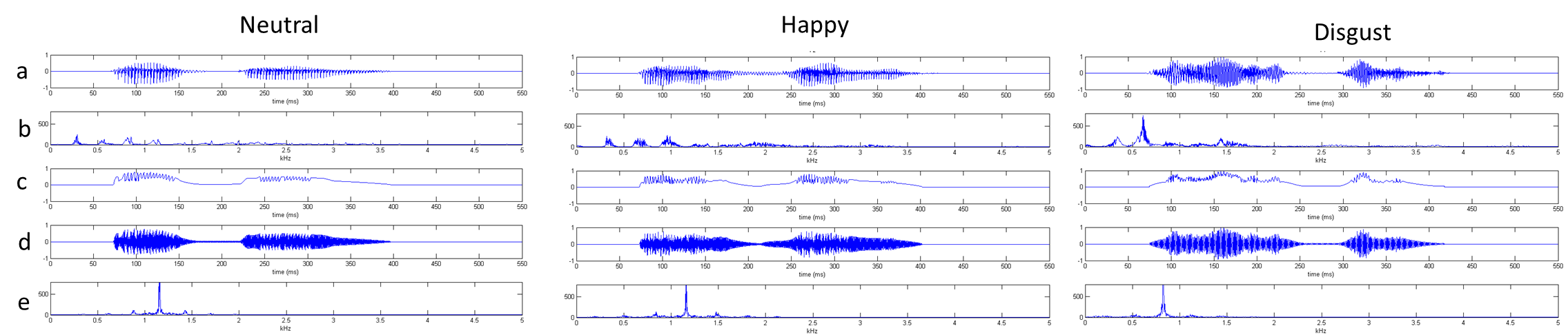


**Figure s3: Sensory ERF across categories.**

Each stimulus type of every category reliably elicits the “N1-P2” complex (red line: disgust, D2; blue line: happy, D1; black line: neutral, S). Nine lines depict three D1s, three D2s and three Ss where three categories are pooled together. For the sake of argument, the grand-average whole-head topography is derived from the average of nine conditions (three D1s, three D2s and three Ss) within an interval from the peak latency of N1m (165ms) to P2m (211ms).

**
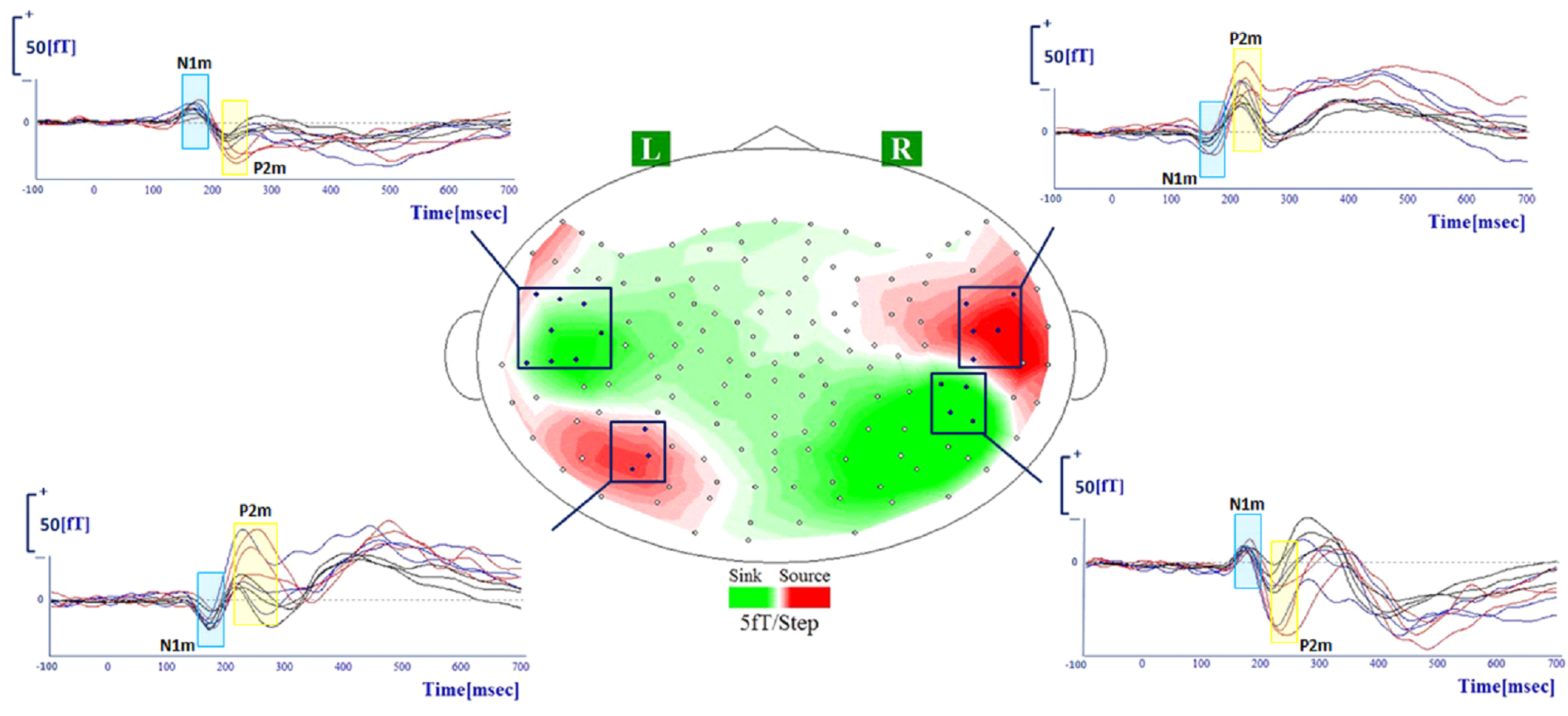
**
